# FAM134B-Mediated ER-phagy Upregulation Attenuates AGEs-Induced Apoptosis and Senescence in Human Nucleus Pulposus Cells

**DOI:** 10.1155/2021/3843145

**Published:** 2021-08-05

**Authors:** Rongjin Luo, Shuai Li, Gaocai Li, Saideng Lu, Weifeng Zhang, Hui Liu, Jie Lei, Liang Ma, Wencan Ke, Zhiwei Liao, Bingjin Wang, Yu Song, Kun Wang, Yukun Zhang, Cao Yang

**Affiliations:** Department of Orthopaedics, Union Hospital, Tongji Medical College, Huazhong University of Science and Technology, Wuhan 430022, China

## Abstract

Previous studies have established the pathogenic role of advanced glycation end products (AGEs) accumulation in intervertebral disc degeneration (IDD). Emerging evidence indicates that ER-phagy serves as a crucial cellular adaptive mechanism during stress conditions. This study is aimed at investigating the role of FAM134B-mediated ER-phagy in human nucleus pulposus (NP) cells upon AGEs treatment and exploring its regulatory mechanisms. We observed that AGEs treatment resulted in significantly increased apoptosis, senescence, and ROS accumulation in human NP cells; meanwhile, the enhanced apoptosis and senescence by AGEs treatment could be partially alleviated with the classic ROS scavenger NAC administration. Furthermore, we confirmed that FAM134B-mediated ER-phagy was activated under AGEs stimulation via ROS pathway. Importantly, it was also found that FAM134B overexpression could efficiently relieve intracellular ROS accumulation, apoptosis, and senescence upon AGEs treatment; conversely, FAM134B knockdown markedly resulted in opposite effects. In conclusion, our data demonstrate that FAM134B-mediated ER-phagy plays a vital role in AGEs-induced apoptosis and senescence through modulating cellular ROS accumulation, and targeting FAM134B-mediated ER-phagy could be a promising therapeutic strategy for IDD treatment.

## 1. Introduction

Intervertebral disc degeneration (IDD) and secondary spine pathological changes such as spinal instability, spinal stenosis, and disc herniation are considered as the leading causes of low back pain, resulting in a substantial burden on the global health care system [1]. Multiple factors have been verified to be associated with the pathogenesis of IDD, including genetic factors, mechanical overloading, nutrition loss, and inflammatory mediators [2–5]. Our previous studies have shown that advanced glycation end products (AGEs) could accumulate in the intervertebral disc with aging and drive the apoptosis of nucleus pulposus (NP) cells and impede its metabolism balance via endoplasmic reticulum (ER) and mitochondria pathway [6–8]. However, the underlying mechanisms are still not fully elucidated.

NP cells are identified as the main cell type resident in the NP tissue, responsible for the synthesis and secretion of the extracellular matrix and maintaining its metabolic balance. The maintenance of an adequate number of functionally active NP cells is a prerequisite for the intervertebral disc to execute its normal physiological activities. It is widely recognized that the degenerated disc is characterized by a decreased number of NP cells and reduced function of residual NP cells [9]. The accumulation of AGEs in the NP during aging process could significantly damage the normal physiological functionalities of intracellular organelles like mitochondria and ER through reactive oxygen species (ROS) generation and calcium mobilization pathway and consequently lead to decreased function or death of NP cells [7, 8]. Although intracellular ROS dyshomeostasis under stress conditions has been well implicated in the pathogenesis of autophagy, senescence, and apoptosis of NP cells, the potential regulatory mechanisms are still not fully explained.

The ER is the central intracellular organelle that responsible for protein synthesis, maturation, and quality control. The protein-folding ability of ER is vulnerably susceptible to genetic and environmental stress, leading to accumulation of unfolded/misfolded proteins in the ER lumen, namely, ER stress, and sustained ER stress can initiate cellular self-destruction procedures [10, 11]. ER-phagy or reticulophagy is a special type of selective autophagy, whereby parts of the ER fragments are engulfed by autophagosomes through specific receptors and then delivered to lysosomal degradation, which in turn attempts to restore cellular energy levels and ER homeostasis [12]. Currently, multiple ER-phagy receptors have been identified in mammals, including FAM134B (RETREG1, reticulophagy regulator 1), RTN3L (reticulon 3 long isoform), SEC62 (SEC62 homolog), CCPG1 (cell-cycle progression gene 1), ATL3 (atlastin 3), and TEX264 (testis-expressed 264), the LIR (LC3-interacting region) domains of which directly recruit phagophores to facilitate ER-phagy [13–18]. FAM134B is the first identified ER-phagy receptor that involves in ER fragments and ER-resident protein clearance in mammalian cells; moreover, dysfunction of FAM134B has been reported to be involved in many diseases, including neuropathy, viral infection, osteoarthritis, and cancer [19–22]. Nevertheless, the role and mechanism of FAM134B and related ER-phagy in the initiation and progress of IDD has not been explored yet.

The purpose of our study was attempted to elucidate the relationship between FAM134B-mediated ER-phagy and apoptosis and senescence under AGEs stimulation. Our studies revealed that AGEs treatment could increase apoptosis, senescence, and FAM134B-mediated ER-phagy through ROS pathway in human NP cells; genetical knockdown and overexpression of FAM134B could increase and reduce cellular ROS generation, apoptosis, and senescence, respectively. Therefore, our findings provide a novel mechanistic insight into the pathogenesis of IDD.

## 2. Materials and Methods

### 2.1. Ethics Statement

Experimental ethics approval for the study was obtained from the Ethics Committee of Tongji Medical College, Huazhong University of Science and Technology (No. S341). Written informed consent was obtained from every donor participated in this study.

### 2.2. Cell Culture and Treatment

The primary human NP cells were isolated from relative undegenerated (Pfirrmann I or II) NP tissues that donated by 3 adolescent idiopathic scoliosis patients (2 males and 1 female, aged 16, 14, and 20 years old, respectively) undergoing spinal deformity correction surgery. The degenerated degree of the corresponding segment was determined by preoperative magnetic resonance imaging according to Pfirrmann classification [23]. Briefly, the freshly harvested human NP tissues were rinsed three times with phosphate buffer saline (PBS, Gibco, Grand Island, NY, USA), minced into 2-3 mm^3^ fragments, and enzymatically digested at 37°C for 8 h in Dulbecco's modified Eagle medium (DMEM/F12, Gibco) supplemented with 0.25 mg/mL type II collagenase (Invitrogen, Carlsbad, CA, USA). Then, the suspension was centrifuged, washed with PBS, and resuspended in DMEM/F12 with 15% fetal bovine serum (FBS; Gibco) and 1% penicillin/streptomycin (Sigma-Aldrich, St. Louis, MO, USA) at 37°C in 5% CO_2_. The NP cell type was confirmed using fluorescently labeled antibody for NP cell markers as described previously [7]. Passage 2 and 3 NP cells were used in subsequent experiments.

In *in vitro* experiments, NP cells were treated with 200 *μ*g/mL AGEs (Abcam, Cambridge, UK) for 0, 6, 12, 24, and 36 h or directly cocultured with AGEs in combination with ROS inhibitor N-acetyl-L-cysteine (NAC, 10 *μ*M, Beyotime, Shanghai, China) for 36 h.

### 2.3. Western Blot Assay

After indicated intervention, cells were harvested using the corresponding protein extraction kit (Beyotime) to lyse and extract protein samples. Proteins were separated through 8-12% sodium dodecyl sulfate- (SDS-) polyacrylamide gel electrophoresis and transferred onto polyvinylidene difluoride (PVDF) membranes (Merck Millipore, Darmstadt, Germany). Next, after blocking with 5% nonfat milk at 25°C for 1 h, the membranes were incubated first with specific primary antibodies (1 : 500-1000) overnight at 4°C and then with the appropriate horseradish peroxidase- (HRP-) labeled secondary antibodies (1 : 2000; Proteintech). Then, membrane bands were visualized by the enhanced chemiluminescence system (Bio-Rad) and quantified with the ImageJ software. Primary antibodies against these molecules were used: p53 (10442-1-AP, Proteintech), p16 (ab151303, Abcam), cleaved caspase 3 (AF7021, Affinity Biosciences,), *β*-actin (66009-1-Ig, Proteintech), FAM134B (21537-1-AP, Proteintech), LC3 (14600-1-AP, Proteintech), and p62 (18420-1-AP, Proteintech).

### 2.4. Cell Proliferation Assay

Cell viability was illustrated using 5-ethynyl-2′-deoxyuridine (EdU) incorporation (C10310-3; Ribobio, Guangzhou, China) according to the manufacturer's instructions. Fluorescence images were captured using a fluorescence microscope (Olympus, BX53, Melville, NY, USA).

### 2.5. Cell Apoptosis Detection

Annexin V-FITC/PI Apoptosis Detection Kit (KeyGEN, Nanjing, China) was used to evaluate apoptosis as described previously [24]. Briefly, after washing with PBS, the cells were labeled with Annexin V–FITC (annexin V) and propidium iodide (PI). After double staining, apoptotic cells were detected via a flow cytometer (BD Biosciences, San Jose, CA, USA). Annexin V+/PI− (early apoptotic) cells and annexin V+/PI+ (late apoptotic) cells were counted.

Terminal deoxynucleotidyl transferase-mediated dUTP nick end labeling (TUNEL) assay was also performed to assess apoptosis. Briefly, after the indicated treatment, cells were washed with PBS and processed with 4% paraformaldehyde for 20 min at 25°C, permeabilized with PBS-0.5% Triton X-100 for 10 min. Then, an in situ cell death detection kit (12156792910; Roche Applied Science, Basel, Switzerland) was used for staining following the manufacturer's protocol. Fluorescence images were acquired through a fluorescence microscope (Olympus).

### 2.6. SA-*β*-gal Staining

SA-*β*-gal assay was performed to detect cell senescence. A SA-*β*-gal staining kit (Beyotime) was used to assess SA-*β*-gal activity according to the manufacturer's instructions. A microscope was used to observe the cells, of which blue-colored cells were counted as SA-*β*-Gal-positive cells. SA-*β*-Gal activity was represented by the percentage of the number of blue cells and the total number of cells.

### 2.7. ROS Measurement

A ROS detection kit (Beyotime) was used to detect the intracellular ROS level according to instructions. Briefly, after indicated treatment, cells were incubated with 10 *μ*M DCFH-DA (2,7-dichlorodihydrofluorescein diacetate dye) in culture media for 30 min. Then, cells were washed with PBS, trypsinized, resuspended in PBS supplemented with FBS, and analyzed for intracellular ROS production by flow cytometry or directly observe fluorescence signal using a fluorescence microscope (Olympus).

### 2.8. Lentivirus and siRNA Transfection

For lentivirus infection, the FAM134B overexpressing lentivirus was designed and constructed by GeneChem (Shanghai, China) using CV084 (Ubi-MCS-SV40-Neomycin) vector. The day before, human NP cells were seeded in 6-well plates at a density of 2 × 10^5^ cells/mL; Then, the cells were infected with lenti-FAM134B or lentivector at a multiplicity of infection (MOI) of 20; transfection efficacy was detected by western blotting after cultured for 72 h. For siRNA-mediated knockdown, control-siRNA and FAM134B-siRNA were purchased from Qijing Biotechnology Co. (Wuhan, China); the corresponding target sequence for RNA interference was 5′-AGCTATCAAAGACCAGTTA. siRNAs were transfected using lipofectamine 2000 (Invitrogen) following the manufacturer's instructions. Cells knocked down for 48 h were followed by the indicated treatment.

### 2.9. Immunofluorescence

NP cells attached to slides were fixed with 4% paraformaldehyde for 20 min, washed three times with PBS, permeabilized with 0.5% Triton X-100 for 15 min, blocked with 2% bovine serum albumin (BSA) for 30 min, and then incubated overnight at 4°C with primary antibodies against p16 (1 : 100, Proteintech), cleaved caspase 3 (1 : 100, CST), FAM134B (1 : 100, Proteintech), and LC3 (1 : 100; Abconal, Wuhan, China). After washed three times with TBST, cells were incubated with CoraLite488 or CoraLite594 conjugated goat anti-rabbit/mouse IgG antibody (1 : 100, Proteintech) for 1 h and labeled with diamidino-2-phenylindole (DAPI; Beyotime) for 5 min and then observed images using a fluorescence microscope (Olympus).

### 2.10. ER-Tracker and Lyso-Tracker Staining

ER-tracker green (40763ES20, Yisheng Biotech, Shanghai, China) and Lyso-tracker red (40739ES50, Yisheng) dyes were used to identify ER-phagy. Briefly, human NP cells were mounted on glass coverslips in a 6-well plate. After the treatment, cells were stained with the recommended concentrations of ER-tracker, Lyso-tracker, and Hoechst 33342 (Beyotime) for 30 min at 37°C, washed three times with PBS, and then observed the fluorescence using a fluorescence microscope (Olympus).

### 2.11. Transmission Electron Microscopy

TEM was used to determine the status of ER positive autophagosome and autolysosome formation. Briefly, after the indicated treatment, NP cells were collected and fixed in a glutaraldehyde and sodium cacodylate solution for 2 h and then fixed with 1% OsO4 for 1.5 h and then stained in 3% aqueous uranyl acetate for 1 h. After washing, specimens were dehydrated with graded ethanol series (50%, 70%, 80%, 90%, 95%, 100%), followed by infiltrating and embedding in epoxy resin (SPI-Chem, #90529-77-4). Ultrathin sections were obtained and stained with saturated uranyl acetate–lead citrate and observed using a transmission electron microscope (Jeol, Tokyo, Japan).

### 2.12. Statistical Analysis

All data were presented as the mean ± standard deviation of at least three independent experiments. Statistical analyses were performed using the GraphPad Prism 8.0 software (La Jolla, CA, USA). Differences between groups were evaluated with Student's *t*-test or one-way ANOVA with post hoc analysis using the Tukey's test. *P* < 0.05 was considered statistically significant.

## 3. Results

### 3.1. AGEs Treatment Promoted Apoptosis and Senescence in Human NP Cells

**Figure 1:**
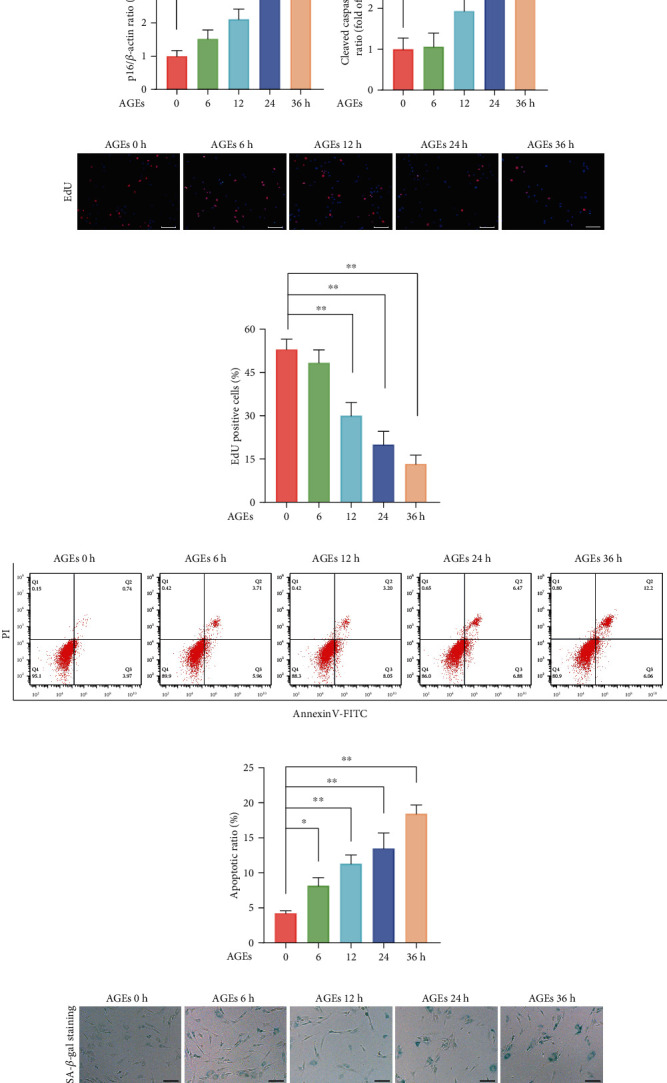
AGEs treatment promoted senescence and apoptosis in human NP cells. The human NP cells were exposed to 200 *μ*g/mL AGEs for different times (0, 6, 12, 24, and 36 h), and 0 h group served as the control. (a–d) Apoptosis and senescence-associated proteins p16, p53, and cleaved caspase 3 were measured using western blot assay, and relative band density was quantified. (e, f) Cell viability was determined using EdU staining combined with DAPI staining for the nuclei, and the positive cell ratio was quantitated, scale bar: 100 *μ*m. (g, h) Representative dot plot images of flow cytometry analysis after labeled with Annexin V-FITC/PI double staining, both early and late apoptosis cells, were quantified. (i, j) Cell senescence was assessed by SA-*β*-gal staining; representative SA-*β*-gal staining images and positive cell ratio were illustrated, scale bar: 50 *μ*m. Data are represented as mean ± SD. ^∗∗^*P* < 0.01, ^∗^*P* < 0.05.

To explore the impact of AGEs on the apoptosis and senescence of human NP cells *in vitro*, a group of NP cells was exposed to AGEs (200 *μ*g/mL) for different times (0, 6, 12, 24, and 36 h). Firstly, apoptosis and senescence-associated proteins p16, p53, and cleaved caspase 3 were determined using western blot assay, as shown in Figures 1(a)–1(d); compared to the control group, the protein expression of p16, p53, and cleaved caspase 3 in the AGEs-treated groups were significantly increased, especially in 24 and 36 h AGEs-treated groups. In addition, EdU staining was also used to assess the cell proliferation ability, as shown in Figures 1(e) and 1(f); the cell viability of human NP cells significantly decreased after AGEs treatment in a time-dependent manner. Moreover, we further employed Annexin V-PI double staining to determine the proapoptotic effects of AGEs on human NP cells, as shown in Figures 1(g) and 1(h); compared to the control group, a clearly higher apoptotic ratio in the AGEs-treated groups was observed. Senescent cells are often concomitant with larger size and higher SA-*β*-gal enzyme activity, as shown in Figures 1(i) and 1(j); the quantification of SA-*β*-gal positive cells in the AGEs-treated groups were robustly higher compared to that in the control group. Thus, our results showed that AGEs treatment could markedly promote apoptosis and senescence in human NP cells.

### 3.2. ROS Pathway Was Involved in AGEs-Induced Apoptosis and Senescence in Human NP Cells

**Figure 2:**
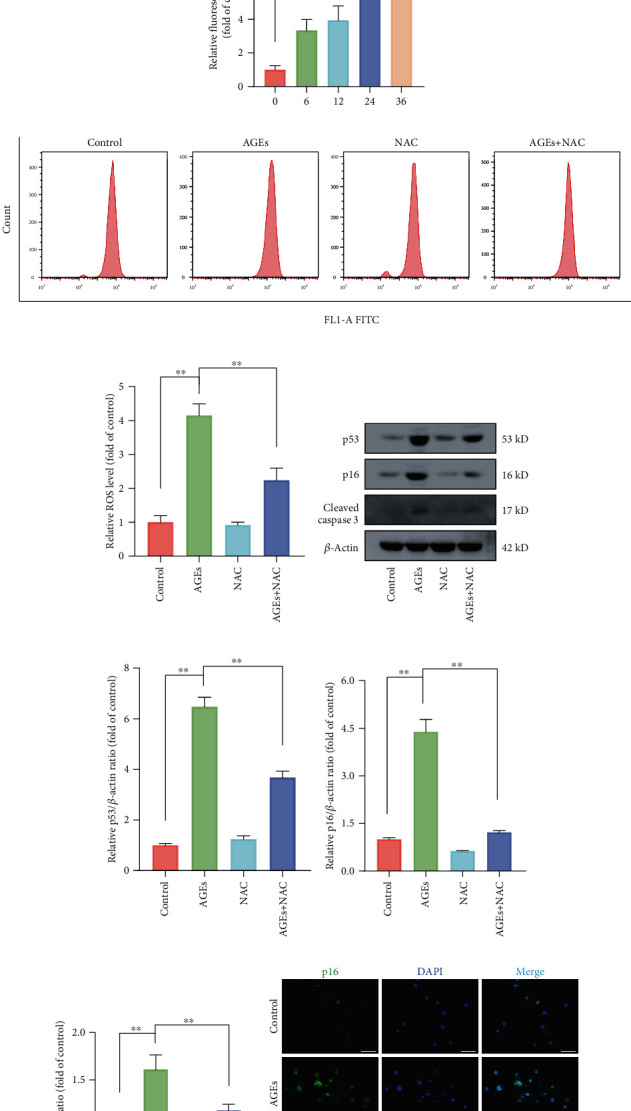
Intracellular ROS accumulation was involved in AGEs-induced senescence and apoptosis. The human NP cells were exposed to 200 *μ*g/mL AGEs for different times (0, 6, 12, 24, and 36 h) or directly cocultured with 200 *μ*g/mL AGEs and ROS inhibitor 10 *μ*M NAC for 36 h. (a, b) The intracellular ROS levels were detected using the fluorescent probe DCFH-DA and measured by flow cytometry. (c, d) After labeled with DCFH-DA fluorescent probe, representative fluorescent images were acquired under a fluorescence microscope, scale bar: 100 *μ*m. (g–j) Representative western blot bands of p16, p53, and cleaved caspase 3 and relative band density were quantified. (k, l) Representative images of immunofluorescence staining for p16 and cleaved caspase-3 in each group, with the relative fluorescence intensity quantified, scale bar: 50 *μ*m. Data are represented as mean ± SD. ^∗∗^*P* < 0.01, ^∗^*P* < 0.05.

Intracellular ROS homeostasis could rapidly change under stress circumstances and function as important messengers involved in cell survival and death. To investigate the relationship between ROS generation and AGEs, we exposed NP cells to AGEs (200 *μ*g/mL) for different times (0, 6, 12, 24, and 36 h) and detected ROS levels using DCFH-DA Assay Kit. As the flow cytometry results shown in Figures 2(a) and 2(b), compared to the control group, AGEs treatment aroused significant time-dependent elevation of intracellular ROS levels and which were consistent with the observation that the AGEs treatment groups showed increased fluorescence intensity compared to the untreated group (Figures 2(c) and 2(d)). NAC is a classical ROS scavenger that reduced intracellular ROS accumulation, as expected, relative to the NAC deficient AGEs-treated group, the NAC existent AGEs-treated group showed clearly lower ROS levels (Figures 2(e) and 2(f)).

To further validate that ROS was involved in AGEs-induced apoptotic and senescent effects, NAC and AGEs were coadministered to human NP cells. As western blot results illustrated in Figures 2(g)–2(j), compared to the corresponding AGEs-treated alone group, protein expression levels of p53, p16, and cleaved caspase 3 were markedly declined in the AGEs plus NAC cotreated group. Similarly, as immunofluorescence results are shown in Figures 2(k) and 2(l). AGEs treatment significantly elevated p16 and cleaved caspase 3 fluorescence intensity relative to that in the control group, while these effects were markedly attenuated in the presence of NAC. Above all, these results suggested that high intracellular ROS level elicited by AGEs was closely associated with the apoptosis and senescence initiation.

### 3.3. FAM134B-Mediatd ER-phagy Was Activated under AGEs Treatment in Human NP Cells

**Figure 3:**
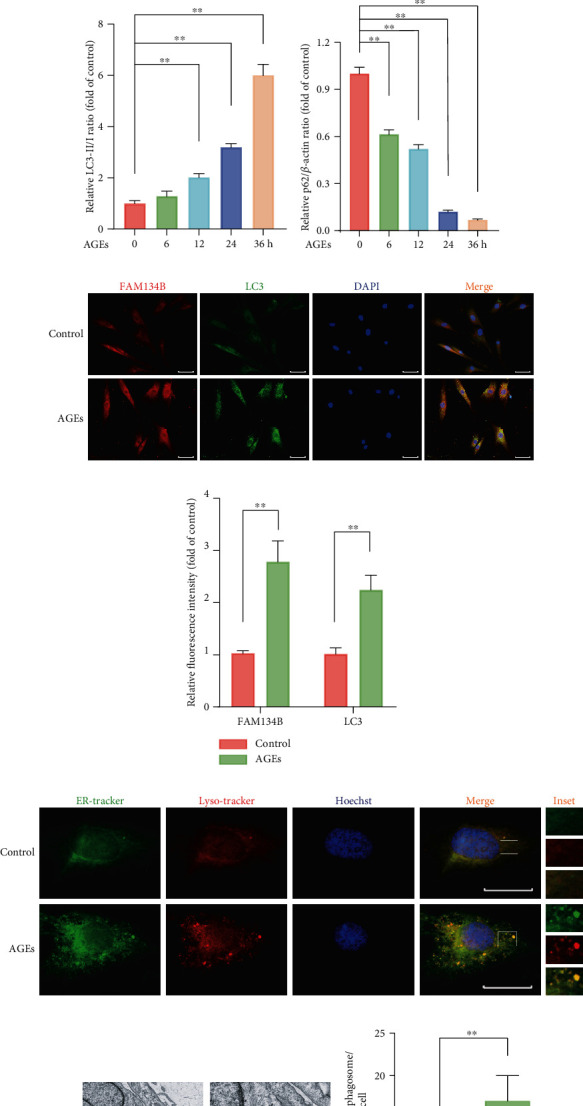
AGEs treatment upregulated FAM134B-mediated ER-phagy in human NP cells. (a–d) The human NP cells were exposed to 200 *μ*g/mL AGEs for different times (0, 6, 12, 24, and 36 h); ER-phagy-associated protein levels of FAM134B, LC3, and p62 were detected by western blot assay, and relative band density was quantified. (e, f) After treated with 200 *μ*g/mL AGEs for 36 h, relative protein expression of FAM134B and LC3 were assessed using immunofluorescence staining; representative images and relative intensity quantification were illustrated, scale bar: 50 *μ*m. (g) ER and lysosome colocalization profile was detected by ER-tracker and Lyso-tracker staining. (h, i) Transmission electron microscopy results for ER positive autophagosomes/autolysosomes (as indicated by black arrow), scale bar: 1 *μ*m and 500 nm. Data are represented as mean ± SD. ^∗∗^*P* < 0.01, ^∗^*P* < 0.05.

FAM134B-mediatd ER-phagy activation is an important regulatory mechanism to solve stress and maintain cellular homeostasis. To investigate the effects of AGEs on FAM134B-related ER-phagy, we exposed NP cells to AGEs (200 *μ*g/mL) for different times (0, 6, 12, 24, and 36 h). FAM134B-related ER-phagy proteins FAM134B, LC3, and p62 were detected by western blot assay, as the results illustrated in Figures 3(a)–3(d); the protein expression profiles of FAM134B and LC3 were robustly upregulated in the AGEs-treated groups compared to that in the control group, along with downregulated autophagy substrate p62 protein levels, indicating the activation of ER-phagy. Meanwhile, as the FAM134B and LC3 fluorescence costaining results shown in Figures 3(e) and 3(f), both the fluorescence intensities of FAM134B and LC3 were significantly enhanced and combined with a higher extent of colocalization upon AGEs treatment. ER and lysosome could be specifically labelled by ER-tracker and Lyso-tracker, respectively; thus, colocalization with the fluorescent signals of ER-tracker green and Lyso-tracker red could be an effective method to determine the occurrence of ER-phagy. As shown in Figure 3(g), compared with the control group, the AGEs-treated group displayed an obvious ER clustering staining pattern, which was partially colocalized with the accumulation of lysosome staining. In addition, we also further monitored the formation of autophagosomes/autolysosomes containing ER fragments under TEM to confirm ER-phagy activation, as shown in Figures 3(h) and 3(i), we observed apparently increased number of autophagosomes/autolysosomes containing ER fragment formation in the AGEs-treated group compared to that in the control group, thereby confirming the occurrence of ER-phagy in human NP cells under AGEs exposition.

### 3.4. ROS Inhibition Partially Attenuated ER-phagy in Human NP Cells Subjected to AGEs Treatment

**Figure 4:**
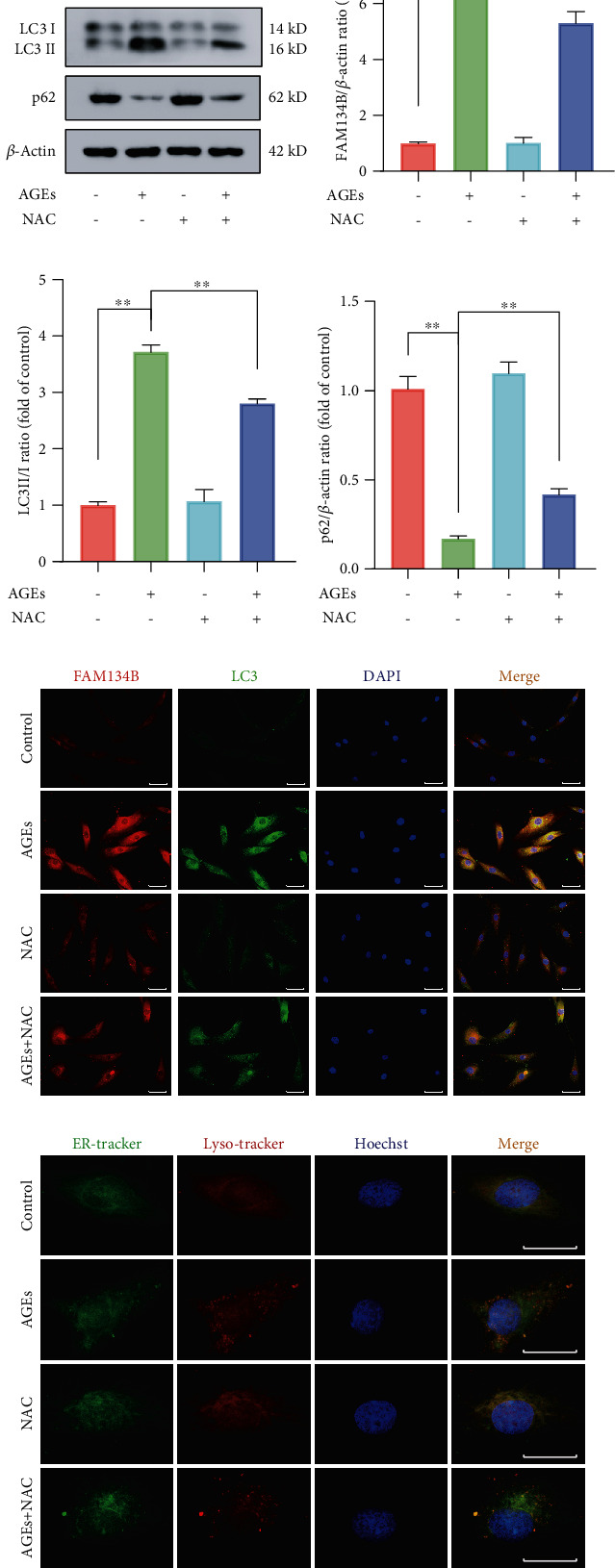
ROS inhibition partially attenuated AGEs-induced ER-phagy activation in human NP cells. The human NP cells were treated with 200 *μ*g/mL AGEs and 10 *μ*M NAC for 36 h. (a–d) ER-phagy-associated protein levels of FAM134B, LC3, and p62 were detected by western blot assay, and the relative band density was quantified. (e) Representative fluorescent images of FAM134B and LC3 were evaluated, and cell nuclei were stained with DAPI. Scale bar: 50 *μ*m. (f) ER and lysosome colocalization was labeled with ER-tracker and Lyso-tracker staining. Data are represented as mean ± SD. ^∗∗^*P* < 0.01, ^∗^*P* < 0.05.

Since both intracellular ROS accumulation and ER-phagy activation upon AGEs treatment were observed, to further test their intrinsic relationship, the ER-phagy activation level under AGEs treatment was determined in the presence of NAC or not. As shown in Figures 4(a)–4(d), the ER-phagy-related protein expression levels were detected using western blot; compared to the corresponding AGEs-treated alone group, the protein expression levels of FAM134B and LC3 were markedly declined in the AGEs plus NAC co-treated group, whereas p62 protein level was slightly increased. Meanwhile, the enhanced fluorescence intensity of FAM134B and LC3 upon AGEs treatment were also significantly attenuated in the presence of NAC administration (Figure 4(c)). In addition, as shown in Figure 4(d), the elevated colocalization of ER and lysosome patterns under AGEs treatment were markedly decreased with NAC cotreatment. Therefore, our findings demonstrated that intracellular ROS accumulation plays a vital role in the process of FAM134B-mediated ER-phagy activation.

### 3.5. Modulation of FAM134B-Mediated ER-phagy Could Regulate Intracellular ROS Level, Apoptosis, and Senescence in AGEs-Treated Human NP Cells

**Figure 5:**
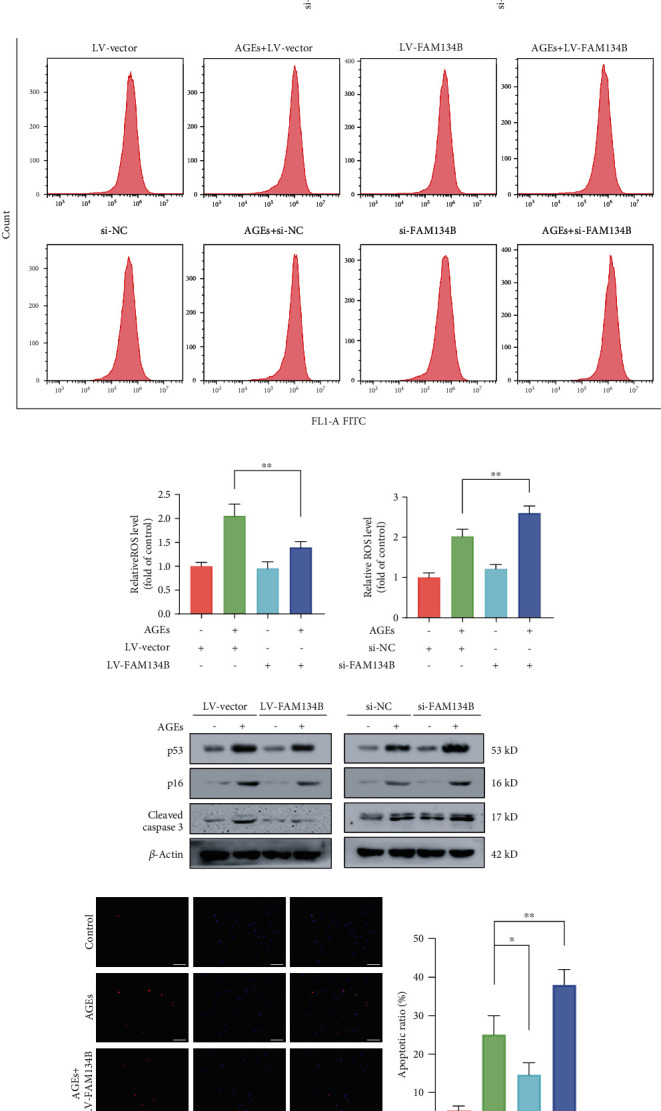
FAM134B-mediated ER-phagy regulated AGEs-induced intracellular ROS accumulation, apoptosis, and senescence in human NP cells. After lentivirus and siRNA transfection were separately conducted for 72 and 48 h, the human NP cells were cultured with 200 *μ*g/mL AGEs for 36 h. (a–d) FAM134B overexpression and knockdown efficacy were verified by western blot assay. (e–g) The intracellular ROS levels were probed using the fluorescent dye DCFH-DA and measured by flow cytometry. (h) Relative protein expression levels of the apoptotic and senescent associated proteins p53, p16, and cleaved caspase3 were evaluated using western blot assay. (i) Cell apoptosis was assessed by TUNEL staining; representative TUNEL immunofluorescent images and apoptotic ratio were quantitated, scale bar: 100 *μ*m. (j) Representative SA-*β*-gal staining images and positive cell ratio were illustrated, scale bar: 50 *μ*m. Data are represented as mean ± SD. ^∗∗^*P* < 0.01, ^∗^*P* < 0.05.

It is classically recognized that FAM134B-meditated ER-phagy acts as an important quality control mechanism in maintaining cellular homeostasis. We next investigated the influence of FAM134B-mediated ER-phagy activation or inhibition on intracellular ROS levels, apoptosis, and senescence by genetically upregulating and downregulating the FAM134B expression. As the western blot results demonstrated in Figures 5(a)–5(d), we efficiently achieved FAM134B overexpression and knockdown by lentiviral transduction and RNA interference, respectively. Subsequently, to determine whether ROS changes in response to FAM134B-mediated ER-phagy, we measured intracellular ROS levels using ROS-sensitive DCFH-DA dye. As the flow cytometry results illustrated in Figures 5(e)–5(g), the ROS level in the AGEs plus lenti-FAM134B group was significantly lower compared with that in the AGEs plus lentivector group, while FAM134B knockdown significantly increased intracellular ROS levels upon AGEs treatment than that noted in the blank knockdown group. Moreover, we next analyzed the effects of FAM134B-mediated ER-phagy on apoptosis and senescence under AGEs treatment. As shown in Figure 5(h), compared to the AGEs plus blank overexpression or knockdown group, FAM134B overexpression markedly attenuated the expression of the apoptotic and senescent associated proteins p53, p16, and cleaved caspase3, while FAM134B knockdown showed the opposite effects. Consistently, as the TUNEL and SA-*β*-gal staining results illustrated in Figures 5(i)–5(l), FAM134B overexpression could alleviate AGEs-induced apoptosis and senescence, while FAM134B suppression could exacerbate the proapoptotic and prosenescent effects of AGEs. Above all, our findings demonstrated that FAM134B-mediated ER-phagy activation could relieve intracellular ROS accumulation, apoptosis, and senescence in human NP cells upon AGEs treatment.

## 4. Discussion

Previous studies have shown that AGEs accumulation in the intervertebral disc along with aging plays a critical role in the pathogenesis of IDD [6–8], and emerging evidence indicates that ER-phagy could serve as a vital intracellular homeostatic regulatory mechanism that directly determines cell functionality and fate under various stress circumstances [19, 25]. However, the role of ER-phagy in AGEs-mediated IDD and its potential mechanisms require further exploration. In the present study, we revealed that FAM134-mediated ER-phagy was markedly activated upon AGEs treatment via ROS pathway in human NP cells, and genetical upregulation and inhibition of FAM134B-mediated ER-phagy could significantly decrease and increase intracellular ROS level, apoptosis, and senescence in NP cells subjected to AGEs stimuli, respectively.

The maintenance of healthy, active, and functional NP is an important prerequisite for the intervertebral disc to properly execute physiological function, which largely depends on the existence of adequate numbers of functionally active NP cells in the disc; thus, numerous studies investigating IDD mainly focus on exploring the underlying causes of abnormal quantity loss and functionality decline of NP cells. Age-related accumulation of AGEs in the intervertebral disc impedes its extracellular matrix synthesis and turnover and ultimately impairs the biomechanical properties of the intervertebral disc and drives the development of IDD [26]. Song et al. found that AGEs accumulation in the disc could impede the anabolic and catabolic balance of NP cells via NLRP3-inflammasome pathway [6]. Furthermore, Song et al. and Luo et al. revealed that AGEs treatment could damage mitochondria redox balance via suppressing Sirt3 function and compromise ER function via disturbing calcium homeostasis and ultimately promote apoptosis [7, 8]. Consistently, we further explored the contribution of AGEs on NP cell apoptosis and senescence in the present study, and the results showed that AGEs treatment restrained cell proliferation, promoted cell apoptosis, and senescence in human NP cells.

ROS represents a group of unstable and highly reactive molecules such as superoxide anions, hydroxyl ions, and hydrogen peroxide, which mainly derived from aerobic metabolism. Physiological quantity of ROS is known to control cellular signal transduction and play roles in cell homeostasis, while excessive ROS produced under stress conditions appear to intervene with normal cell physiology and even lead to cell death [27]. Excessive ROS accumulation has been implicated in the pathogenesis of IVD degeneration [28, 29]. Furthermore, Xiang et al. and Kang et al. revealed that modulation of the cellular ROS level through antioxidant administration could efficiently mitigate oxidative stress-induced NP cell death [30, 31]. NAC, a ROS scavenger, was used to confirm the role of ROS in the proapoptotic and prosenescent effects of AGEs in the present study; as expected, we observed that the administration of NAC significantly attenuated AGEs-induced ROS burst, apoptosis, and senescence of NP cells.

ER-phagy, referred to the selective degradation of the ER by autophagy, is emerging as a critical regulator of cell homeostasis and function. Notably, the highly selective process is largely achieved through selective receptor that interact with autophagosome-associated LC3 and subsequently cargo ER fragments and ER-resident proteins for lysosomal degradation [32]. To date, six reticulophagy receptors have been identified in mammals: FAM134B, RTN3L, SEC62, CCPG1, ATL3, and TEX264, among which FAM134B was the first one to be identified. Although the intrinsic relationship between ER-phagy and classical macroautophagy is still not fully understood, several studies found that disruption of the interaction of FAM134B with LC3 by mutation or deletion of the LIR amino acid sequence failed to induce ER-phagy [17]. Moreover, the two major autophagy regulators, ATG5 and BECN1, are essential for ER fragmentation and degradation induced by FAM134B overexpression, suggesting that FAM134B-induced ER-phagy depends on the core macroautophagy machinery [33, 34]. ER-phagy is considered a stress-induced response mechanism to maintain cellular homeostasis and to promote cell survival; ER-phagy defection has been validated to be associated with multiple human pathologies, including infectious, neurodegenerative diseases, aging, and cancer [35]. Indeed, in the present study, there are significantly increased autophagosomes containing layered membrane structures, and much more ER and lysosome colocalization clustering was observed upon AGEs treatment, suggesting the occurrence of ER-phagy in NP cells under AGEs stimuli.

Furthermore, ER-phagy is emerging recognized as an alternative ER quality and quantity control regulatory mechanism via delivering excess ER fragments and ER-resident protein for lysosomal degradation, which is critical for cellular homeostasis and adaptation to variable environments. Strikingly, ER-phagy is substantially enhanced when encountering stress conditions such as starvation and contributes to resolve ER stress and reestablish ER homeostasis [16]. It is confirmed that FAM134B-mediated ER-phagy could restrict viral replication via eliminating ER-associated viral proteins, while suppression or knockdown of FAM134B could facilitate viral replication [36]. Additionally, a higher survival rate was observed in breast cancer patients with higher FAM134B expression [37]. Notably, the relationship between FAM134B-mediated ER-phagy and apoptosis is pretty complicated; ER-phagy could either act as a protective mechanism against apoptosis via restoring excessive ER stress [38, 39] or exhibit ER-phagy-dependent cell death via accelerating ER degradation and impairs ER homeostasis to trigger ER stress [21, 40]; these evidence may indicate the possibility that the adaptive capability of ER-phagy is limited; when encountered severe conditions overwhelming the regulatory capacity of ER-phagy, the protective ER-phagy may switch to facilitate cell death. Accordingly, we detected whether ER-phagy upregulation exerts a protective effect against AGEs stimuli in NP cells, and the results showed that the enhanced expression apoptotic and senescent associated proteins p53, p16, and cleaved caspase 3, and intracellular ROS levels were significantly decreased with FAM134B overexpression, indicating that FAM134B-mediated ER-phagy plays a critical protective role in defending AGEs-induced cell damage.

There are still several shortcomings in our study. Firstly, despite the facts that our research has suggested a crucial role of FAM134B-mediated ER-phagy activation in balancing intracellular ROS level and promoting cell survival, while the specific molecular mechanisms remain to be further studied. Moreover, we only focused on the typical ER-phagy receptor FAM134B in this study, whether other types of ER-phagy receptors are involved in this process was not explored. In addition, the current study lacks *in vivo* experimental and clinical validation.

Collectively, we demonstrated that FAM134B-mediated ER-phagy activation plays a crucial role in protecting against AGEs-induced intracellular ROS accumulation and cell injury in human NP cells, and targeting FAM134B-mediated ER-phagy may be a potentially effective therapeutic strategy for IDD.

## Data Availability

The data supporting the findings of this study are included in the article.

## Abbreviations

AGEs: Advanced glycation end products
IDD: Intervertebral disc degeneration
NP: Nucleus pulposus
ROS: Reactive oxygen species
ER: Endoplasmic reticulum
FAM134B: RETREG1, reticulophagy regulator 1
RTN3: Reticulon 3 long isoform
SEC62: SEC62 homolog
CCPG1: Cell-cycle progression gene 1
ATL3: Atlastin 3
TEX264: Testis-expressed 264
EdU: 5-Ethynyl-2′-deoxyuridine
TUNEL: Terminal deoxynucleotidyl transferase-mediated dUTP nick end labeling
NAC: N-Acetyl-L-cysteine
SA-*β*-gal: Senescence-associated *β*-galactosidase.
